# Supplementary material for: Isolation and Characterization of Germline Stem Cells in Protogynous Hermaphroditic Monopterus albus
Source: Int J Mol Sci. 2022 May 24;23(11):5861. doi: 10.3390/ijms23115861 (PMC9180834; doi:10.3390/ijms23115861)
Supplement: Supplementary file 1 [file ijms-23-05861-s001.zip › ijms-1701276.pptx]

## Slide 1
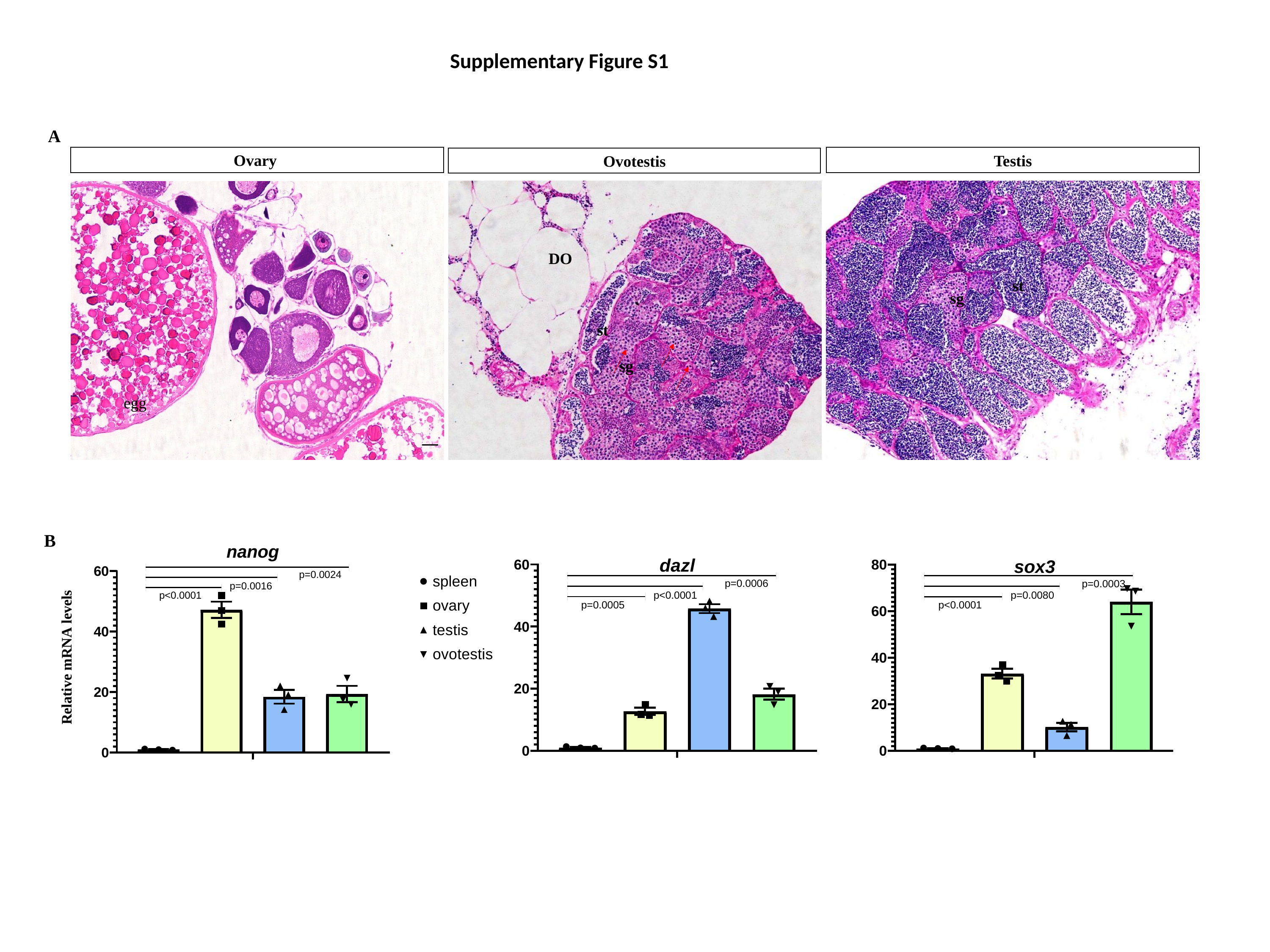

Supplementary Figure S1
A
Ovary
Testis
Ovotestis
DO
st
sg
st
sg
egg
B

## Slide 2
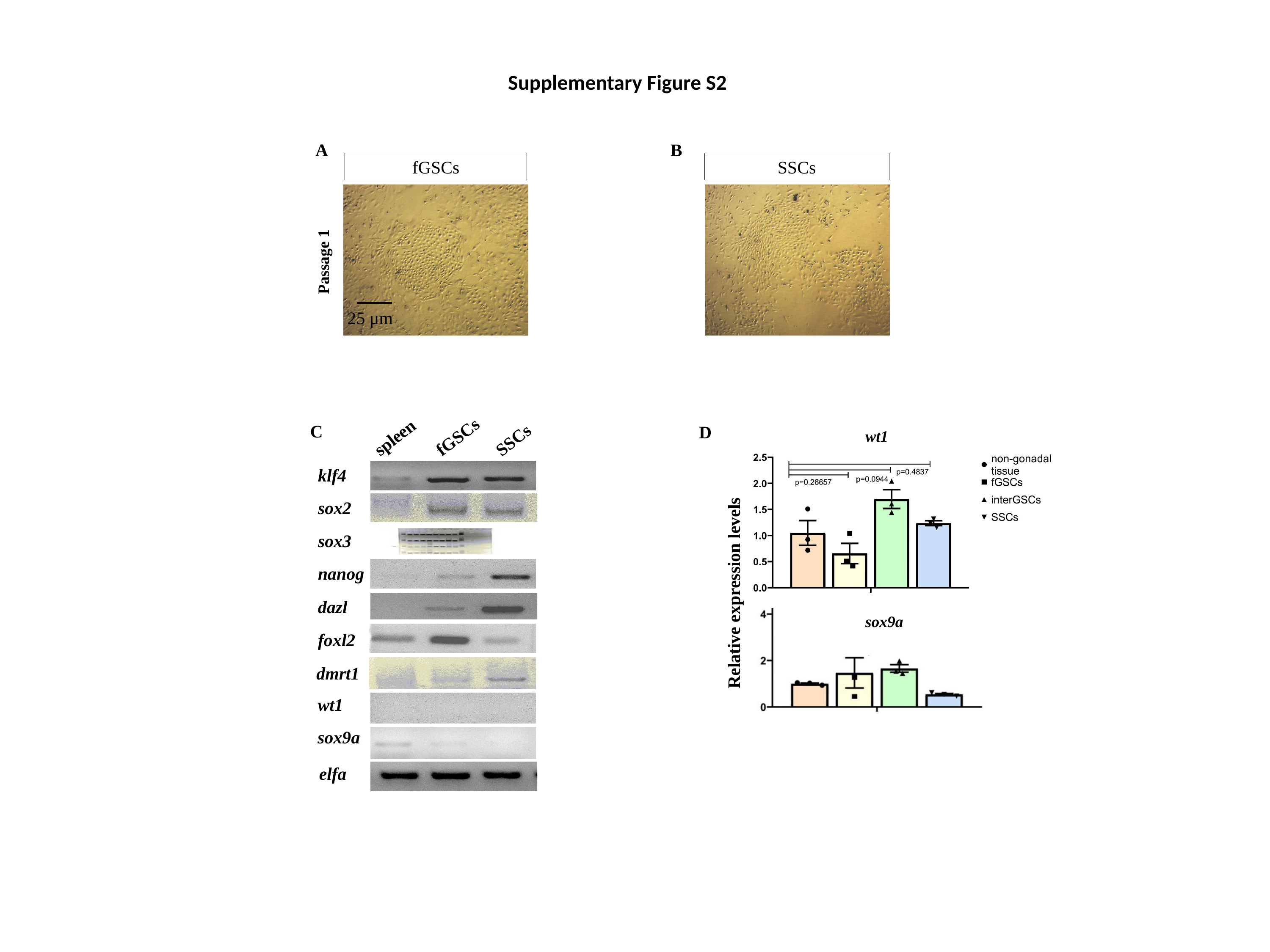

Supplementary Figure S2
A
B
fGSCs
SSCs
Passage 1
25 μm
SSCs
spleen
fGSCs
C
klf4
sox2
sox3
nanog
dazl
foxl2
dmrt1
wt1
sox9a
elfa
D
wt1
sox9a
Relative expression levels

## Slide 3
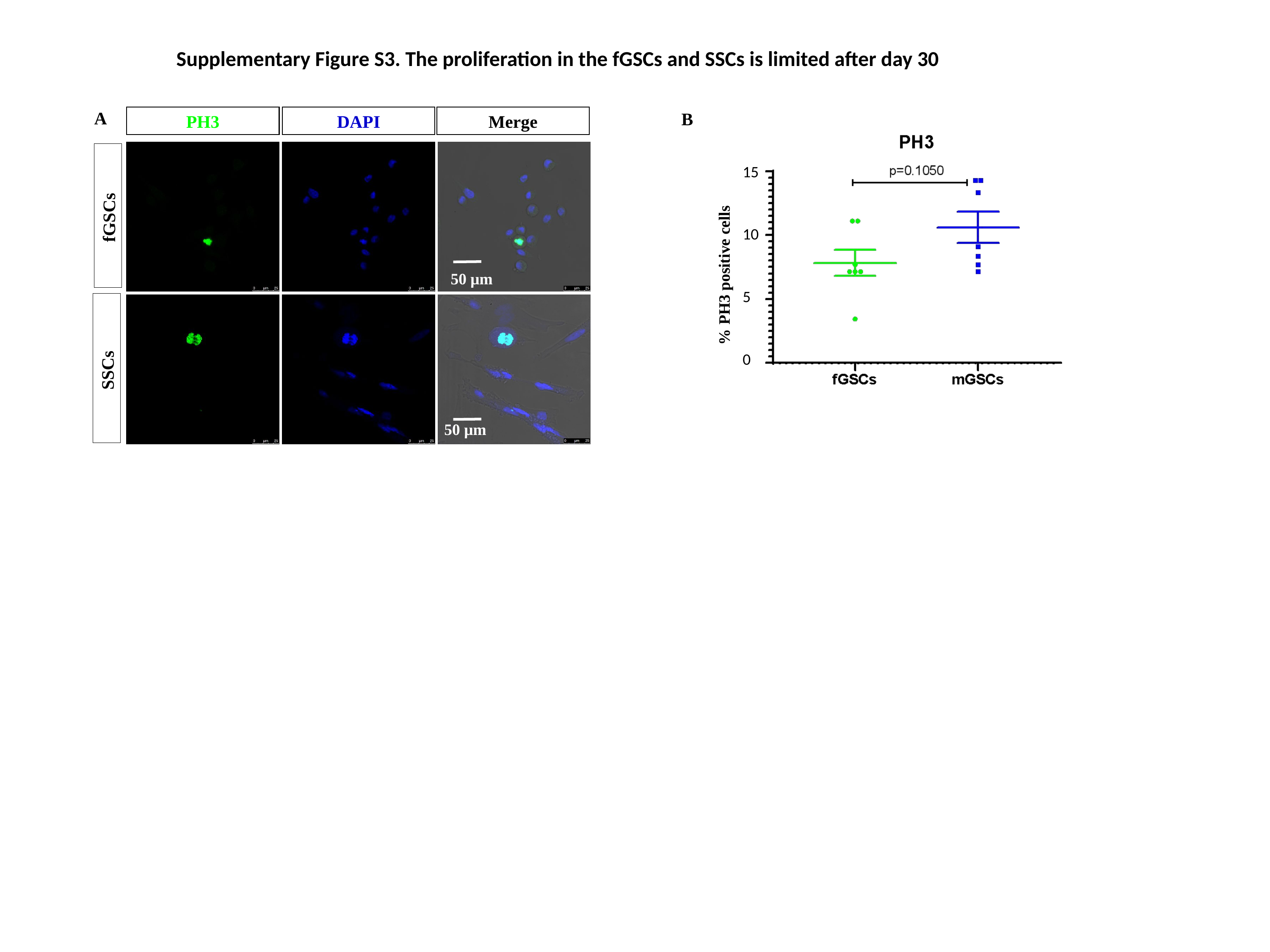

Supplementary Figure S3. The proliferation in the fGSCs and SSCs is limited after day 30
A
PH3
DAPI
Merge
50 μm
fGSCs
50 μm
SSCs
B
15
10
5
0
% PH3 positive cells

## Slide 4
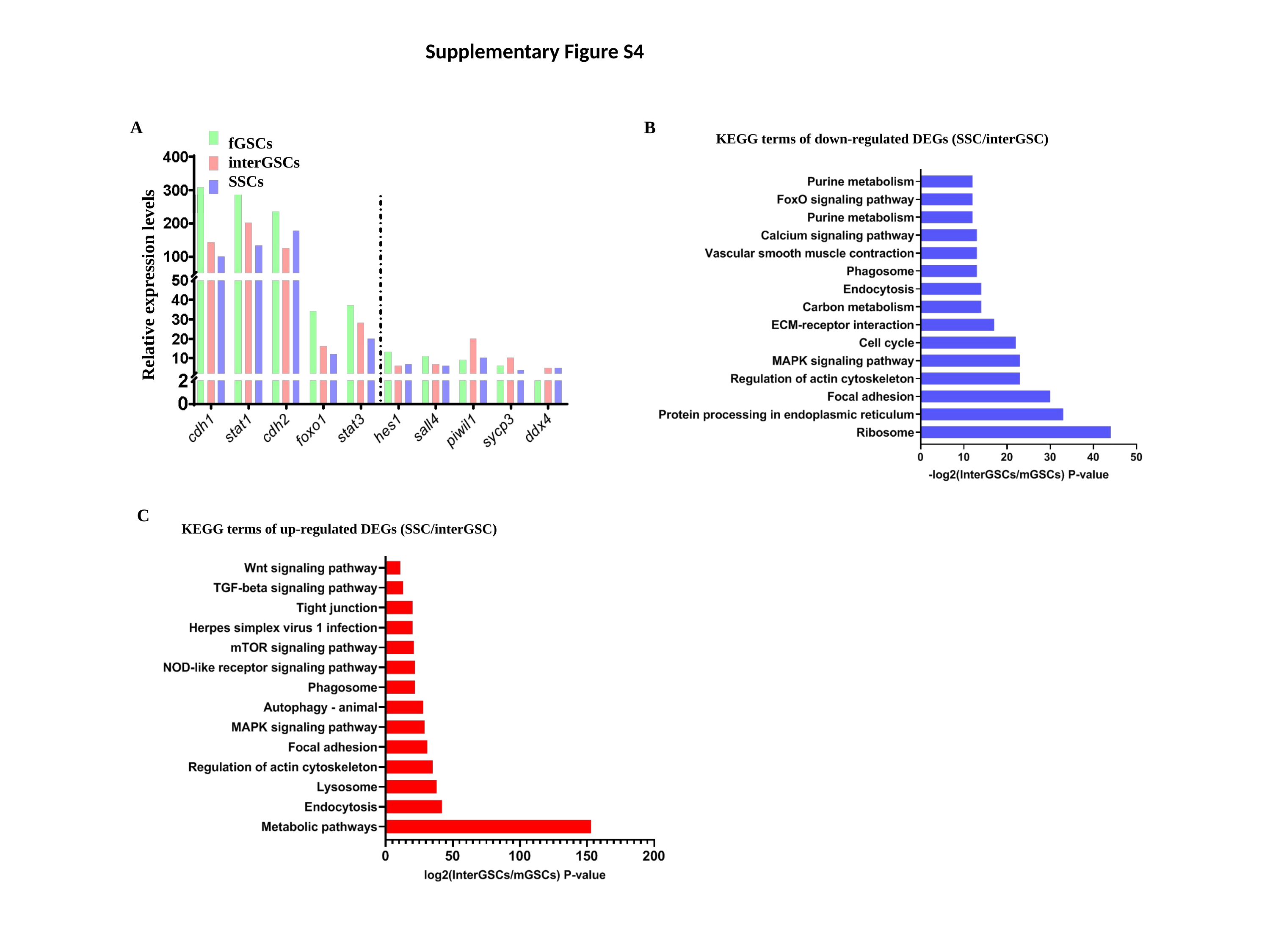

Supplementary Figure S4
A
fGSCs
interGSCs
SSCs
Relative expression levels
B
KEGG terms of down-regulated DEGs (SSC/interGSC)
C
KEGG terms of up-regulated DEGs (SSC/interGSC)

## Slide 5
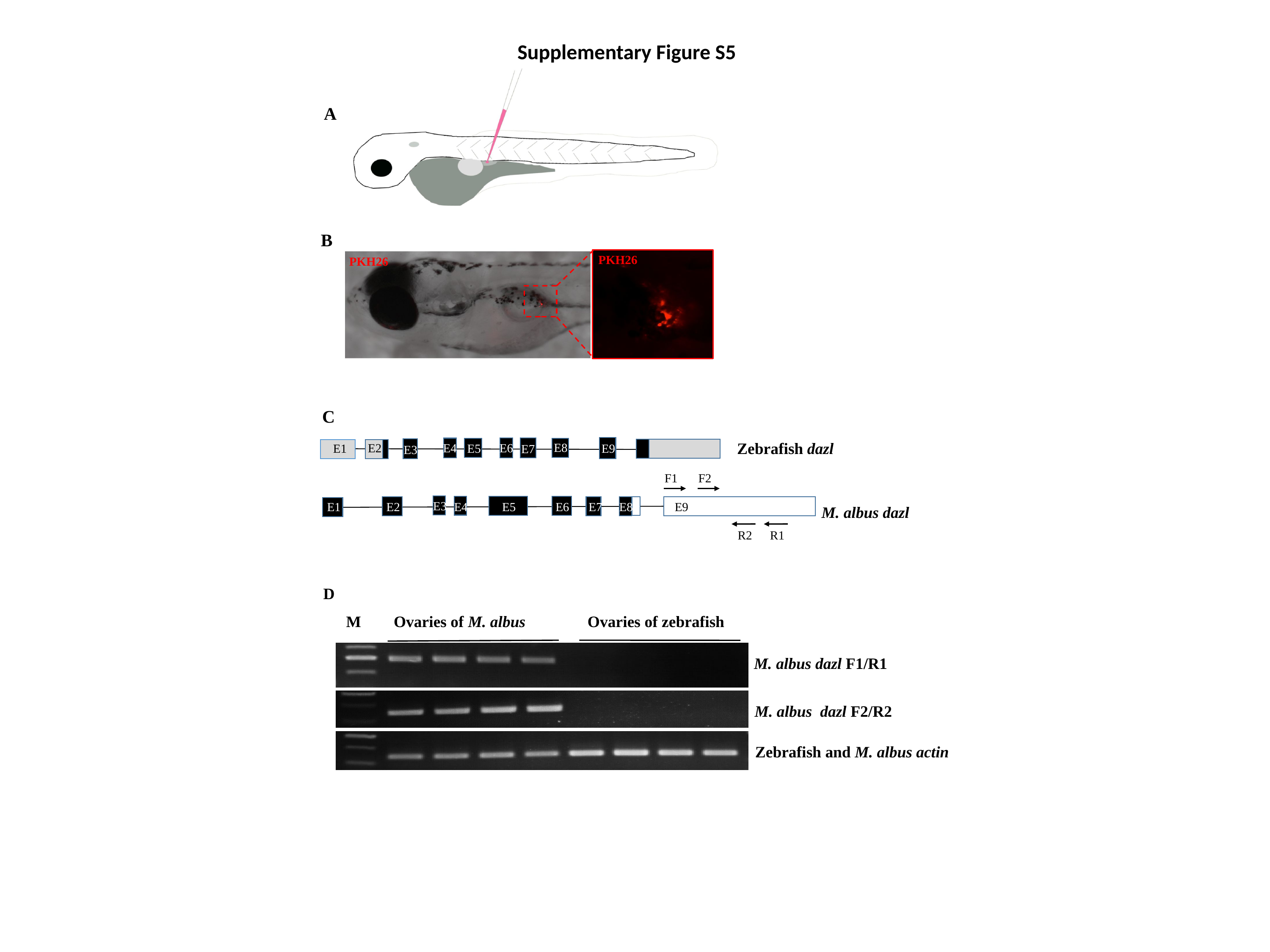

Supplementary Figure S5
A
B
 PKH26
 PKH26
C
Zebrafish dazl
E8
E6
E2
E4
E5
E1
E9
E7
E3
E10
F1
F2
E3
E6
E7
E9
E1
E2
E4
E5
E8
R2
R1
M. albus dazl
D
M
Ovaries of M. albus
Ovaries of zebrafish
M. albus dazl F1/R1
M. albus dazl F2/R2
Zebrafish and M. albus actin
